# High-Fat Diet Promotes Adipogenesis in Offspring Female Rats Induced by Perinatal Exposure to 4-Nonylphenol

**DOI:** 10.1155/2023/6540585

**Published:** 2023-06-23

**Authors:** Hongyu Zhang, Weiran Ke, Xi Chen, Yu Han, Yan Xiong, Feng Zhu, Yang Xiang, Rong Yan, Hongbo Cai, Shunmei Huang, Xiaoyu Ke

**Affiliations:** ^1^School of Biological and Pharmaceutical Engineering, Wuhan Polytechnic University, Wuhan 430030, China; ^2^College of Life Sciences and Oceanography, Shenzhen University, Shenzhen 518060, China; ^3^Department of Nosocomial Infection Management, Tongji Hospital of Tongji Medical College, Huazhong University of Science and Technology, Wuhan 430030, China; ^4^Department of Geriatrics, First Affiliated Hospital, School of Medicine, Zhejiang University, Hangzhou 310003, China; ^5^Emergency Department and Intensive Care Unit, Tongji Hospital of Tongji Medical College, Huazhong University of Science and Technology, Wuhan 430030, China

## Abstract

**Background:**

Both high-fat diet (HFD) and 4-nonylphenol (4-NP) could affect fat formation in adipose tissue individually. We investigated whether HFD promote abnormal adipose tissue formation caused by early exposure to 4-NP in life and preliminarily explore the possible mechanisms involved.

**Methods:**

The first-generation rats were treated with HFD on postnatal day after pregnant rats exposure to 5 ug/kg/day 4-NP. Then, the second generation rats started to only receive normal diet without 4-NP or HFD. We analyzed organ coefficient and histopathology of fat tissues, biochemical index, and gene level involved in lipid metabolism in female offspring rats.

**Results:**

HFD and 4-NP interaction synergistically increased birth weight, body weight, and organ coefficients of adipose tissue in offspring female rats. HFD accelerately aggravated abnormal lipid metabolism and increased the adipocyte mean areas around the uterus of the offspring female rats induced by prenatal exposure to 4-NP. HFD also facilitate the regulation of gene expression involved lipid metabolism in offspring female rats induced by perinatal exposure to 4-NP, even passed on to the second generation of female rats. Moreover, HFD and 4-NP interaction synergistically declined the gene and protein expression of estrogen receptor (ER) in the adipose tissue of second-generation female rats.

**Conclusion:**

HFD and 4-NP synergistically regulate the expression of lipid metabolism genes in adipose tissue of F2 female rats and promote adipose tissue generation, leading to obesity in offspring rats, which is closely related to low expression of ER. Therefore, ER genes and proteins may be involved in the synergistic effect of HFD and 4-NP.

## 1. Background

Obesity as the most common chronic disease seriously endangers human health. And the incidence rate of obesity is on the rise worldwide, but the exact cause and pathogenesis of obesity are still unclear. High-fat diet is increasingly considered as an indispensable reason for the higher obesity rate with the change of diet structure in modern society. Many epidemiological studies have shown that the incidence rate of obesity is positively correlated with the average amount of fat in the diet [1]. Moreover, accumulating evidences suggest that the 4-NP pollution is very serious in foods, especially in the animal foods with high-fat content [2, 3]. HFD exacerbate the chance of 4-NP which could be stored in HFD and exposed to the human beings. 4-NP may affect the adipocyte differentiation and, subsequently, result in obesity as one kind of typical environmental endocrine disrupting chemicals (EDCs) [4].

Increasing evidence suggests that environmental stimuli during the perinatal period have a prolonged effect on the offspring, even leading to obesity or other chronic adult diseases. Some researches showed that maternal exposure to the abnormal environment changed the metabolic phenotype in the first generation of rats, and this same effect could occur in the next or several generations [5]. The intrauterine hyperglycemia environment may damage the glucose tolerance and normal insulin levels of offspring rats [5]. Our previous research found that HFD and NP simultaneously caused abnormal expression of fatty acid synthesis genes in the liver tissue of second-generation rats and had a certain synergistic effect, ultimately leading to fat accumulation in the liver tissue of offspring rats [6]. Moreover, perinatal exposure to the 4-NP might increase the organ coefficients of adipose tissue and the serum cholesterol level in offspring rats [7]. This genetic change, even if nonexposed to the environmental stimuli, would also be passed to the next generation or several generations [8]. Clinical evidence suggests that excessive intake of estrogen substances is closely related to obesity. Estrogen directly acts on estrogen receptors, regulates the expression of downstream lipid metabolism genes, affects adipose tissue formation, and leads to excessive accumulation of adipose tissue in the body [9]. Many genes, such as fatty acid synthase (Fas), nonesterified fatty acids (NEFA), and peroxisome proliferator-activated receptors (PPARs), regulate the generation of adipose tissue and play a crucial role in obesity, but their mechanisms are still unclear. We aimed to determine whether the HFD promote the adiogenesis in offspring female rats induced by perinatal exposure to 4-NP, whether such synergistic effect could be passed on to the next generation and the potential mechanisms involved.

## 2. Methods

### 2.1. Animal Studies

Twenty-four wild virgin Wistar rats were purchased from Beijing Vital Rivers (Beijing, China) and were kept under specific pathogen-free conditions in the Experimental Animal Centre of Tongji Medical College, Huazhong University of Science and Technology. All the animals approved by the Ethics Committee of Tongji Medical College were treated according to the Guidelines of the National Institutes of Health for Animal Care and Use.

### 2.2. Maintenance and Treatment with the 4-NP and the HFD

**Figure 1:**
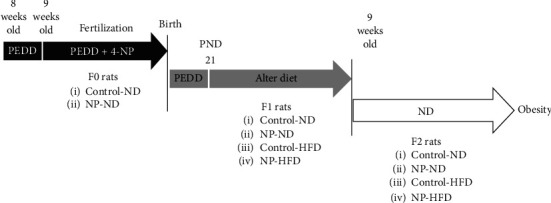
Experimental design and intervention methods.

Phytoestrogen deficient diet (PEDD, Shanghai Laboratory Animal Center, Shanghai, China, which contains 13.21% fat, 27.18% protein, and 59.61% carbohydrates, with the energy of 14.39 kJ/g kcal/g) was used to feed with Virgin Wistar rats (F0) from the 11 days before being caged to postnatal day (PND) 21, which would exclude the intervention effect by other estrogenic substance in the normal diet. One male rat and two female rats were caged in one cage to obtain the first-generation rats (F1). The time that the sperm-positive smear was observed under a microscope was defined as gestation day (GD). It was randomly divided into two groups (8 pregnant rats for each group): the control-ND group treated with olive oil and the NP-5-ND group treated with NP (5 *μ*g/kg/day), both of which received a normal diet (Figure 1). It was divided into four groups for F1 male or female rats with similar body weight on PND 21: control ND group (16 rats) and NP-5-ND group (16 rats), fed with normal diet; control-HFD group (16 rats) and NP-5-HFD (16 rats), receiving high-fat diet (28.53% fat, 22.33% protein, 49.14% carbohydrates, with energy of 17.36 kJ/g) (Figure 1).

Male F1 rats (9 weeks old) were mated with the female F1 rats (9 weeks old) in the four groups (8 rats per group) to obtain the second-generation rats (F2) which were given a normal diet after weaning (Figure 1). F1 female rats at 23 weeks and F2 female rats at 13 weeks were anesthetized with pentobarbital sodium (intraperitonealed with 50 mg/kg pentobarbital sodium) and euthanasiaed by cervical dislocation. The serum was collected and stored in a refrigerator at −80°C. The perirenal adipose tissue and periuterine adipose tissue were separated rapidly, washed by PBS, dried by the filter paper, and weighted to figure out the organ coefficient.

### 2.3. Biochemical Assays

The serum levels of triglycerides (TG) and blood-glucose (GLU) were determined with an automatic blood analyzer (Mindray BS-200; Shenzhen, China). Serum nonesterified free fatty acids (NEFA) were measured by colorimetric assay (Jiancheng Biological Institute, Nanjing, China). Leptin and adiponectin concentrations were determined by the ultrasensitive mouse leptin and adiponectin enzyme-linked immunosorbent assay (ELISA) (Linco Research, Millipore, Billerica, MA).

### 2.4. Detection of the Serum 4-NP in F1 and F2 Female Offspring

Serum samples were collected from the PND21 offspring in F1 female rats and F2 female rats. The serum levels of the 4-NP were determined with a liquid chromatography-tandem mass spectrometry (LC-MS/MS) according to the method previously reported [10].

### 2.5. Hematoxylin and Eosin (H/E) Staining for the Size of Adipose Cells

The number and size of adipose cells were visualised by HE staining of fat tissue that had been fixed with 4% paraformaldehyde solution. Under a light microscope (Olympus CKX41, Japan), the quantity and areas of 120 adipocytes were measured in each captured image. Photographs were taken by using a Zeiss Axiocam Camera (Carl Zeiss, Gottingen, Germany) and assembled in Photoshop 6.0 (Adobe Systems, Mountain View, CA).

### 2.6. RNA Sequencing

The total RNA was isolated from the perigonadal adipose tissue in F2 generation rats by using the E.Z.N.A. total RNA kit (Omega, Norcross, GA, USA). The quality and quantity were detected using a NanoDrop ND-1000 spectrophotometer (Thermo Scientific). RNA libraries were made according to the manufacturer's protocol of the TruSeq RNA sample preparation kit (Illumina, San Diego, CA, USA) and analyzed with the Genome Analyzer II sequencing system in the BGI Technology Services Co., Ltd., China. Differentially expressed genes were selected according to *P* < 0.05 or a fold change greater than 2.

### 2.7. Real-Time Reverse Transcription PCR

Total RNA was isolated from the fat tissue by using the E.Z.N.A. total RNA kit (Omega, Norcross, GA, USA). One-step RT-PCR with real-time detection was conducted with the SYBR Green Real-Time RT-PCR Master Mix (Toyobo, Osaka, Japan). The mRNA levels of Lpl, Fas, Srebp-1, Ppar-*γ*, ER*α*, and 36B4 were detected with the PCR conditions (22). Relative gene expression was calculated by the 2^−△△Ct^ method with 36B4 as an endogenous reference gene.

### 2.8. Western Blotting

Aliquots of proteins from fatty tissue lysates were electrophoresed on 10% (w/v) polyacrylamide gels and transferred onto nitrocellulose membranes (Schleicher & Schuell, Kassel, Germany). HRP-conjugated secondary antibodies (GB23404, Service bio Biotechnology Inc., China) were applied after being probed with antibodies for ER*α* (GB11026, Servicebio Biotechnology Inc., China) or *β*-actin (GB12001, Servicebio Biotechnology Inc., China).

### 2.9. Statistical Analysis

Statistical analyses were performed by using SPSS 18.0. Comparisons between the two groups were analyzed via two independent samples *t*-test. One-way ANOVA or two-way ANOVA was applied with Bonferroni's test when different groups were compared. *P* value <0.05 was considered statistically significant.

## 3. Results

### 3.1. HFD and 4-NP Interaction Has Catalytic Role on the Birth Weight of F2 Female Rats

**Figure 2:**
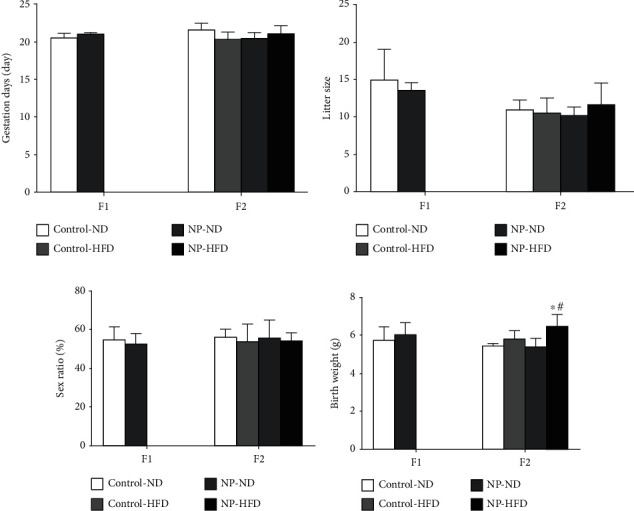
The effect of HFD and 4-NP on gestation days, little size, and sex ratio of offspring rats of F1 and F2 offspring rats. HFD and 4-NP interaction had a synergistic effect on the birth weight of F2 offspring rats. ^∗^*P* < 0.05 vs. control; ^#^synergistic effect existed.

The serum 4-NP in postnatal day 21 of F1 and F2 female rats were detected as zero. Recent reports have demonstrated that early life exposure to endocrine disrupting chemical was correlated with the increased body weight [11]. Therefore, we detected the effect of the synergistic effect of HFD and 4-NP on the changes of gestation days, the litter size, sex ratio, and birth weight. HFD or 4-NP alone had no synergistic effect on gestation days, litter size, sex ratio, and birth weight of offspring rats. But HFD and 4-NP interaction had a synergistic effect on the birth weight of F2 offspring rats (*P* < 0.05), with no significant changes on gestation days, litter size, or sex ratio of offspring rats (Figure 2).

### 3.2. HFD Promote an Increase of the Body Weight and Organ Coefficients of Adipose Tissue in the Offspring Female Rats Induced by Perinatal Exposure to 4-NP

4-NP or HFD alone could significantly increase the body weights and organ coefficient in F1 and F2 offspring female rats (*P* < 0.05). Moreover, 4-NP and HFD interaction could synergistically increase the body weight and organ coefficient of adipose tissue in F1 and F2 offspring female rats (*P* < 0.05), as shown in Table 1.

### 3.3. HFD and 4-NP Interaction Aggravatingly Affect on the Disorder of Glucose and Lipid Metabolism in Offspring Female Rats

HFD alone upregulated the levels of leptin, triglyceride (TG), glucose (Glu), and nonesterified fatty acids (NEFA) in F1 and F2 female rats and downregulated the levels of adiponectin in F1 and F2 female rats (*P* < 0.05), while 4-NP could upregulate the levels of leptin, TG, and Glu of F1 and F2 female rats and downregulate the levels of adiponectin of F1 and F2 female rats (*P* < 0.05). In addition, HFD and 4-NP interaction could synergistically enhance the levels of leptin, TG, Glu, and NEFA of F1 and F2 female rats (*P* < 0.05), as shown in Table 2.

### 3.4. HFD Accelerately Increase the Adipocyte Mean Area in Offspring Female Rats Induced by Perinatal Exposure to 4-NP

**Figure 3:**
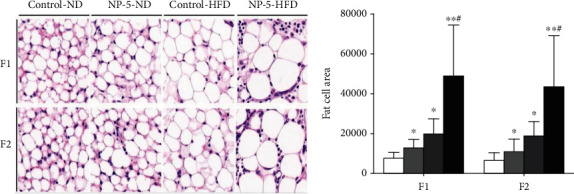
The pathomorphology change of adipose tissues around the uterus in F1 and F2 female rats (×40). HFD and 4-NP had a synergistic effect on adipocyte mean area around the uterus in offspring female rats. Note: ^∗^*P* < 0.05 vs. control; ^∗∗^*P* < 0.01 vs. control; #synergistic effect existed.

The adipocyte mean area around the uterus in the HFD or 4-NP group of F1 and F2 female rats was bigger, compared to the adipocyte mean area of the control group. HFD and 4-NP synergistically increased the adipocyte mean area around the uterus in offspring female rats. Obviously, HFD and 4-NP had higher degree of inflammatory aggregation and fibrosis, compared to the control group (Figure 3).

### 3.5. HFD Facilitate the Regulation of Gene Expression Involved Lipid Metabolism in Offspring Female Rats Induced by Perinatal Exposure to 4-NP

**Figure 4:**
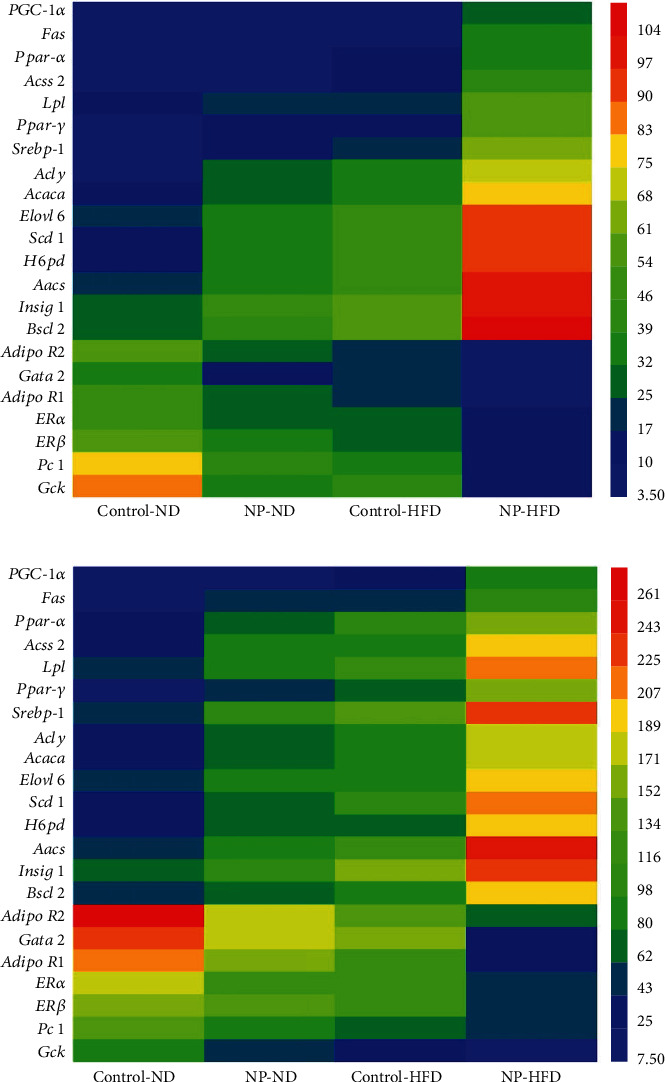
The effect of HFD and 4-NP on the gene expression of adipose tissue in F1 and F2 female rats. (a) HFD and 4-NP interaction synergistically regulated gene expression involved lipid metabolism in F1 female rats. (b) HFD and 4-NP interaction synergistically regulated gene expression involved lipid metabolism in F2 female rats.

Signature DEG comparison and ontological analyses of the RNA-seq data highlight the gene differential expression involved in glucolipid metabolism in F1 and F2 female rats. HFD or 4-NP treated alone significantly upregulated the gene expression involved fatty acid synthesis, and HFD and 4-NP interaction synergistically upregulated gene expression involved fatty acid synthesis in adipose tissue in F1 and F2 female rats, such as the peroxisome proliferator-activated receptor-*γ* coactivator-1*α* (*PGC-1α*), fatty acid synthase (*Fas*), peroxisome proliferator-activated receptor *α* (*Ppar-α*), acetyl-CoA synthetase 2 (*Acss 2*), Lpl, peroxisome proliferator-activated receptor-*γ* (*Ppar γ*), Srebp-1, ATP-citrate lyase (*Acly*), acetyl coenzyme A carboxylase *α* (*Acaca*), long-chain fatty acids family member 6 (*Elovl 6*), stearoyl-CoA desaturase 1 (*Scd 1*), hexose-6-phosphate dehydrogenase (*H6pd*), acetoacetyl-CoA synthetase (*Aacs*), insulin-induced gene (*insig1*), and the Berardinelli-Seip congenital lipodystrophy 2 (*Bscl 2*) (Figure 4).

Moreover, HFD or 4-NP treated alone significantly downregulated the RNA level of some genes in adipose tissue in F1 and F2 female rats, for example, adiponectin receptor 2 (*Adipo R2*), GATA binding protein 2 (*Gata 2*), adiponectin receptor 1 (*Adipo R1*), plasma cell membrane glycoprotein-1 (*PC-1*), and glucokinase (*Gck*). HFD and 4-NP interaction synergistically downregulated the RNA level of these genes in adipose tissue in F1 and F2 female rats (Figure 4).

**Figure 5:**
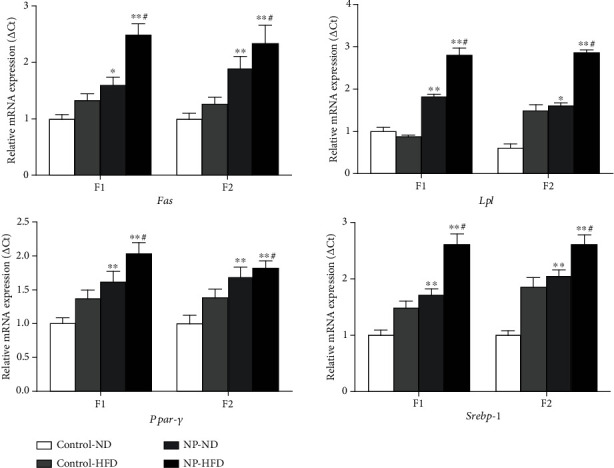
The effect of HFD and 4-NP on the mRNA expression of adipose tissue in F1 and F2 female rats. HFD and 4-NP interaction synergistically upregulated mRNA level of Fas, Lpl, *Ppar-γ*, and *Srebp*-1 in F1 and F2 female rats. ^∗^*P* < 0.05 vs. control-ND; ^∗∗^*P* < 0.01 vs. control-ND; #synergistic effect existed.

In addition, RT-PCR assays were applied to confirm the mRNA expression involved in fatty acid synthesis in adipose tissue in offspring female rats. It was shown that 4-NP treated alone significantly upregulated the mRNA level of Fas, Lpl, *Ppar-γ*, and *Srebp*-1 in offspring female rats. HFD and 4-NP interaction synergistically upregulated mRNA level of Fas, Lpl, *Ppar-γ*, and *Srebp*-1 in F1 and F2 female rats (Figure 5).

### 3.6. HFD and 4-NP Interaction Synergistically Reduce the Gene and Protein Expression of ER in the Adipose Tissue of F2 Female Rats

**Figure 6:**
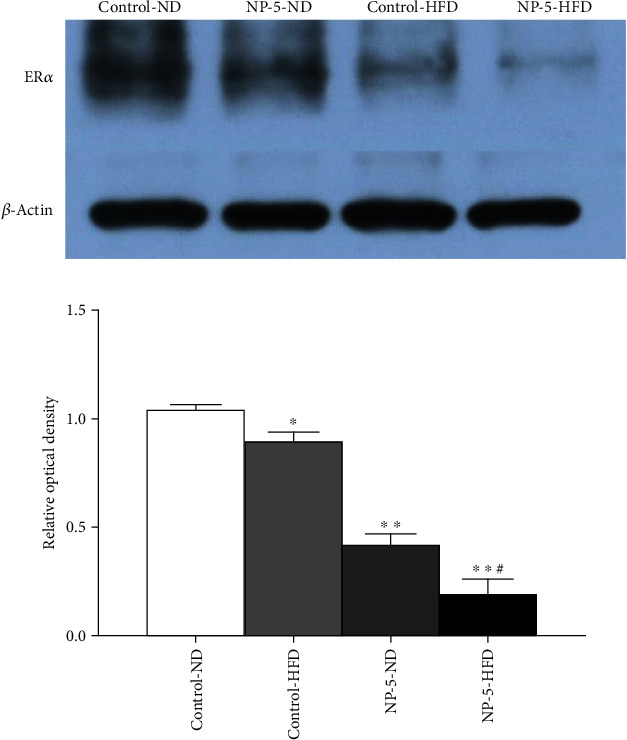
The effect of HFD and 4-NP on the expression of ER*α* in the fatty tissue around the uterus of F2 female rats. HFD and 4-NP interaction synergistically reduce the gene and protein expression of ER*α* in adipose tissue of F2 female rats. ^∗^*P* < 0.05 vs. control-ND; ^∗∗^*P* < 0.01 vs. control-ND; #synergistic effect existed.

It is shown in Figure 4 that HFD or 4-NP alone significantly decreased the gene expression level of ER in offspring female rats, and HFD and 4-NP interaction synergistically reduce the gene expression of ER in adipose tissue of offspring female rats. We also detect the synergistic effects of HFD and 4-NP on the protein expression of ER*α* in the fatty tissues of F2 generation rats. The result has shown that HFD or 4-NP alone significantly decreased the protein expression level of ER*α* in female F2 rats. HFD and 4-NP interaction synergistically reduce the protein expression of ER*α* in adipose tissue of F2 female rats (Figure 6).

## 4. Discussion

Human and zoological researches show that the metabolic phenotype resulted from matrilineal exposure to the abnormal environment can be inherited by the next generation. With the increasing incidence of obesity, a large number of scientific researches have focused on the correlation between the maternal environment and obesity and the long-term effect on the offspring [12]. Pregnancy exposed to endocrine-disrupting chemicals could produce transgenetic effects by epigenetic modification [13, 14]. We studied whether a high-fat diet promotes the adipogenesis in offspring female rats induced by perinatal exposure to the typical endocrine disruptor 4-nonylphenol. It was confirmed in our experiment that the level of serum 4-NP in postnatal day 21 of offspring rats was detected as zero due to the given low dose of 4-NP and the biological metabolism. HFD or 4-NP alone had no significant effect on gestation days, litter size, sex ratio, and birth weight of offspring rats, but HFD and 4-NP interaction had synergistic effect on the birth weight of F2 offspring rats. Moreover, HFD or 4-NP alone had significant effect on body weight and organ coefficient of offspring rats, and HFD and 4-NP interaction had synergistic effect on body weight and organ coefficient of offspring rats.

As we all know, the most important biological effects of leptin as a kind of hormone mediated by neuropeptide-containing neurons and neuropeptide receptors in the hypothalamus could inhibit appetite, reduce energy intake, increase energy consumption, and inhibit fat synthesis [15]. Adiponectin as an insulin-sensitizing hormone is an important regulator in the regulatory network of lipid metabolism and blood glucose homeostasis [16]. And adiponectin treatment significantly reduced the content of blood triglyceride and low-density lipoprotein and increased the content of high-density lipoprotein [17]. Free fatty acids, also known as nonesterified fatty acids (NEFA), are the intermediate products of fat metabolism and the important metabolic substrates of energy metabolism in cells of the body. The concentration of NEFA in serum is closely related to obesity [18]. Our results also indicated that HFD or 4-NP alone upregulated the levels of leptin, TG, Glu, and NEFA in offspring female rats and downregulated the levels of adiponectin in offspring female rats: HFD and 4-NP interaction could synergistically enhance the levels of leptin, TG, Glu, and NEFA of offspring female rats. Moreover, HFD and 4-NP had a synergistic effect on adipocyte mean area around the uterus in offspring female rats. In other words, HFD synergistically promoted abnormal lipid metabolism and pathomorphology change of adipose tissues around the uterus in offspring female rats induced by perinatal exposure perinatal exposure to 4-NP, which maybe genetically altered and passed on to the next generation rats.

Abnormal expression of fatty acid synthesis genes alters the formation of adipose tissue and even leads to obesity. Elevated NEFA concentrations in obesity are thought to arise from an increased adipose tissue mass [19]. Fatty acid synthase (Fas) encodes the key enzymes for fatty acid synthesis which is related to the accumulation of adipose tissue [20]. Fas is also involved in the activation of Peroxisome proliferator-activated receptors (PPARs) which is the nuclear receptor superfamily and could regulate the metabolism of fatty acids at the transcriptional level [21]. Sterol regulatory element-binding protein- (Srebp-) 1 is also now well established as a key transcription factor for the regulation of lipogenic enzyme genes such as FAS [22]. ATP-citrate lyase (Acly) fuels pivotal biochemical reactions such as the synthesis of fatty acids [23]. Stearoyl-coenzyme A desaturase 1 (SCD1) is a central regulator of fuel metabolism and catalyzes the synthesis of monounsaturated fatty acids (MUFAs) [24]. There are many master-regulator of lipid syntheses such as acetyl-CoA carboxylase (Acaca), acetoacetyl-CoA synthetase (AACS), acetyl-CoA synthetase 2 (ACSS2), insulin-induced gene (Insigs), and Berardinelli-Seip's congenital lipodystrophy 2 (Bscl2) [25–29]. Recently, new data suggest that peroxisome proliferator-activated receptor-*γ* coactivator-1*α* (PGC-1*α*) plays an important role in the regulation of mitochondrial biogenesis along with other genes involved in adipose tissue [30]. Lipoprotein lipase (Lpl) as an important lipogenetic gene catalyzes triglyceride into glycerin and fatty acids which are ingested into adipocytes in adipose tissue [31]. Inactive cortisol is converted into active cortisol under the action of enzyme 11-*β* hydroxysteroid dehydrogenase type 1 (HSD11B1), thereby promoting the occurrence of obesity [32]. Our experiment showed that HFD or 4-NP treated alone significantly upregulated the gene expression involved in fatty acid synthesis, and HFD and 4-NP interaction synergistically upregulated gene expression involved fatty acid synthesis in adipose tissue in offspring female rats, such as PGC-1*α*, Fas, Ppar-*α*, Acss 2, Lpl, Ppar *γ*, Srebp-1, Acly, Acaca, Elovl 6, Scd 1, H6pd, Aacs, Insig1, and Bscl 2, which induced the proliferation and differentiation of adipocytes. In addition, it was confirmed that 4-NP treated alone significantly upregulated the mRNA level of Fas, Lpl, *Ppar-γ*, and *Srebp*-1 in offspring female rats, even HFD and 4-NP interaction synergistically upregulated mRNA level of Fas, Lpl, *Ppar-γ*, and *Srebp*-1 in offspring female rats.

Numerous experiments and clinical observations have shown that adiponectin levels in the circulation of obese patients are significantly reduced, and adiponectin affects fat formation through binding to adiponectin receptors (AdipoR1 and AdipoR2) [33]. Defective GATA-2 expression regulates adipocyte differentiation through molecular control of the preadipocyte-adipocyte transition, which is associated with obesity [34]. A polymorphism in plasma cell membrane glycoprotein 1 (PC-1) has been demonstrated to be associated with insulin resistance in obesity [35]. Glucokinase (Gck) serves as a critical element to the regulatory feedback loop that interconnects the major insulin target tissues such as adipose tissue [36]. Our results showed HFD or 4-NP treated alone significantly downregulated the expression of genes such as Adipo R2, GATA-2, PC-1, and Gck. HFD and 4-NP interaction synergistically downregulated the expression level of the gene in adipose tissue in offspring female rats, such as Adipo R2, GATA-2, PC-1, and Gck. In other words, prenatal exposure to 4-NP and HFD synergistically promote lipid metabolism and even fat accumulation in adipose tissues through regulating gene expression level, which might pass on to the next generation of rats. However, the specific molecular mechanism underlying this passage genetic effect has been unclear.

ER-alpha and ER-beta in adipose tissues are importantly involved in lipid homeostasis which may have critical implications for risk factors associated with obesity [37]. 4-NP induced estrogen response element-mediated activity via ER*α* [38]. Whether ER played a vital role in the synergistic effect of HFD plus 4-NP on the induction of obesity in offspring rats. It was shown in our experiment that HFD or 4-NP alone significantly decreased the gene level of ER-alpha and ER-beta in offspring female rats. HFD and 4-NP interaction synergistically reduce the gene level of ER-alpha and ER-beta in adipose tissue of offspring female rats. Moreover, HFD or 4-NP alone significantly decreased the protein expression level of ER-alpha in female F2 rats. HFD and 4-NP interaction synergistically reduce the protein level of ER-alpha in adipose tissue of F2 female rats. In another words, exposure to 4-NP in the early stages of life altered the expression level of genes involved in fat accumulation in F1 rats and synergistically with later high-fat diet interventions on F2 offspring, resulting in abnormal gene expression in the adipose tissue of F2 rats. New research now shows that ER-alpha controls metabolism in white and brown adipocytes by affecting the expression of Polg1 (which encodes the mtDNA polymerase *γ*-subunit) and by influencing mitochondrial remodelling [39]. The results provide new ideas for obesity in human. The daily exposure of perinatal pregnant women to 4-NP and HPD in food might synergistically induced adipogenesis in adipose tissue of offspring female rats, which could be passed on to the next generation, seriously increasing the risk of obesity in offspring.

## 5. Conclusion

Low levels of 4-NP pollution in the environment and a high-fat diet lifestyle synergistically alter gene expression in adipose tissue of two generations of rats and even cause adipose tissue accumulation, leading to obesity. 4-NP and high-fat diet also synergistically act on ER in rat adipose tissue, causing an imbalance in the downstream energy regulatory system and abnormal expression of genes involved in lipid metabolism. Abnormal expression of ER gene may induce genetic effects of obesity in offspring rats. The role of ER in adipose tissue may provide more effective therapeutic targets for obese patients.

## Figures and Tables

**Table 1:** The effect of HFD and NP on body weight and adipose tissue coefficient of F1 (23 weeks old) and F2 (13 weeks old) female rats.

		ND		HFD		*P* value		
		Control-ND	NP-5-ND	Control-HFD	NP-5-HFD	Diet	Nonylphenol	*D* × *N*
Body weight (g)	F1	231 ± 8	290 ± 13	283 ± 11	357 ± 27	<0.05	<0.05	<0.05
	F2	205 ± 7	244 ± 12	261 ± 9	331 ± 17	<0.05	<0.05	<0.05
Organ coefficient (%)	F1	2.95 ± 0.35	4.36 ± 0.15	4.49 ± 0.36	5.59 ± 0.75	<0.05	<0.05	<0.05
	F2	2.31 ± 0.40	4.06 ± 0.58	4.20 ± 0.61	5.37 ± 0.69	<0.05	<0.05	<0.05

Note: *n* = 7 − 8 rats/group. The data represent the mean ± SD.

**Table 2:** The effect of HFD and NP exposure on blood biochemical parameters in F1 and F2 female rats.

		ND		HFD		P-value		
		Control-ND	NP-5-ND	Control-HFD	NP-5-HFD	Diet	Nonylphenol	*D* × *N*
Leptin (nmol/L)	F1	6.17 ± 0.37	8.06 ± 0.28	8.64 ± 1.18	10.92 ± 0.73	<0.05	<0.05	<0.05
	F2	6.64 ± 0.28	7.99 ± 0.38	8.25 ± 0.44	11.31 ± 0.79	<0.05	<0.05	<0.05
Adiponectin(nmol/L)	F1	174 ± 6	150 ± 9	142 ± 9	99 ± 8	<0.05	<0.05	<0.05
	F2	191 ± 9	157 ± 8	145 ± 6	105 ± 11	<0.05	<0.05	<0.05
TG (mg/dl)		64 ± 9	74 ± 5	72 ± 4	99 ± 7	<0.05	<0.05	<0.05
	F2	65 ± 5	68 ± 7	71 ± 4	78 ± 3	<0.05	<0.05	<0.05
Glu (mg/dl)	F1	121 ± 16	137 ± 14	144 ± 17	164 ± 5	<0.05	<0.05	<0.05
	F2	60 ± 13	80 ± 14	91 ± 13	122 ± 9	<0.05	<0.05	<0.05
NEFA (*μ*g/L)	F1	0.60 ± 0.78	0.63 ± 0.98	0.78 ± 0.17	1.39 ± 0.14	<0.05	>0.05	<0.01
	F2	0.60 ± 0.76	0.65 ± 0.12	1.58 ± 0.11	2.59 ± 0.09	<0.01	>0.05	<0.05

Note: *n* = 8 rats/group. The data represent the mean ± SD.

## Data Availability

The data that support the findings of this study are available from the corresponding author upon reasonable request.

## References

[1] Perez G. S., Cordeiro G. D. S., Santos L. S., Espírito-Santo D. D. A., Boaventura G. T., Barreto-Medeiros J. M. (2021). Does a high-fat diet-induced obesity model brown adipose tissue thermogenesis? A systematic review. *Archives of Medical Science: AMS*.

[2] Carabajal M. D., Arancibia J. A., Escandar G. M. (2019). Excitation-emission fluorescence-kinetic third-order/four-way data: determination of bisphenol a and nonylphenol in food-contact plastics. *Talanta*.

[3] Chung S. (2021). The development of isomer-specific analysis of branched 4-nonylphenol in food for dietary exposure - a critical review of analytical methods and occurrence in foodstuffs. *Food Additives & Contaminants. Part A, Chemistry, Analysis, Control, Exposure & Risk Assessment*.

[4] Hao C. J., Cheng X. J., Xia H. F., Ma X. (2012). The endocrine disruptor 4-nonylphenol promotes adipocyte differentiation and induces obesity in mice. *Cellular Physiology and Biochemistry*.

[5] Kaspar D., Hastreiter S., Irmler M., Hrabé de Angelis M., Beckers J. (2020). Nutrition and its role in epigenetic inheritance of obesity and diabetes across generations. *Mammalian Genome*.

[6] Zhang H., Song C., Yan R., Cai H., Zhou Y., Ke X. (2021). High-fat diet accelerate hepatic fatty acids synthesis in offspring male rats induced by perinatal exposure to nonylphenol. *BMC Pharmacology and Toxicology*.

[7] Zhang H. Y., Xue W. Y., Li Y. Y. (2014). Perinatal exposure to 4-nonylphenol affects adipogenesis in first and second generation rats offspring. *Toxicology Letters*.

[8] Ng S. F., Lin R. C. Y., Laybutt D. R., Barres R., Owens J. A., Morris M. J. (2010). Chronic high-fat diet in fathers programs *β*-cell dysfunction in female rat offspring. *Nature*.

[9] Rubinow K. B. (2017). Estrogens and body weight regulation in men. *Advances in Experimental Medicine and Biology*.

[10] Xiao Q., Li Y., Ouyang H., Xu P., Wu D. (2006). High-performance liquid chromatographic analysis of bisphenol a and 4-nonylphenol in serum, liver and testis tissues after oral administration to rats and its application to toxicokinetic study. *Journal of Chromatography. B, Analytical Technologies in the Biomedical and Life Sciences*.

[11] Braun J. M. (2017). Early-life exposure to EDCs: role in childhood obesity and neurodevelopment. *Nature Reviews. Endocrinology*.

[12] Rodgers A., Sferruzzi-Perri A. N. (2021). Developmental programming of offspring adipose tissue biology and obesity risk. *International Journal of Obesity*.

[13] Lu X., Fraszczyk E., van der Meer T. (2020). An epigenome-wide association study identifies multiple DNA methylation markers of exposure to endocrine disruptors. *Environment International*.

[14] Stel J., Legler J. (2015). The role of epigenetics in the latent effects of early life exposure to obesogenic endocrine disrupting chemicals. *Endocrinology*.

[15] Obradovic M., Sudar-Milovanovic E., Soskic S. (2021). Leptin and obesity: role and clinical implication. *Frontiers in Endocrinology*.

[16] Balsan G. A., Vieira J. L. . C., Oliveira A. M. ., Portal V. L. (2015). Relationship between adiponectin, obesity and insulin resistance. *Revista da Associação Médica Brasileira*.

[17] Samant N. P., Gupta G. L. (2021). Adiponectin: a potential target for obesity-associated Alzheimer's disease. *Metabolic Brain Disease*.

[18] Hierons S. J., Marsh J. S., Wu D., Blindauer C. A., Stewart A. J. (2021). The interplay between non-esterified fatty acids and plasma zinc and its influence on thrombotic risk in obesity and type 2 diabetes. *International Journal of Molecular Sciences*.

[19] Karpe F., Dickmann J. R., Frayn K. N. (2011). Fatty acids, obesity, and insulin resistance: time for a reevaluation. *Diabetes*.

[20] Batchuluun B., Pinkosky S. L., Steinberg G. R. (2022). Lipogenesis inhibitors: therapeutic opportunities and challenges. *Nature Reviews. Drug Discovery*.

[21] Porcuna J., Minguez-Martinez J., Ricote M. (2021). The PPAR*α* and PPAR*γ* epigenetic landscape in cancer and immune and metabolic disorders. *International Journal of Molecular Sciences*.

[22] Sekiya M., Yahagi N., Matsuzaka T. (2007). SREBP-1-independent regulation of lipogenic gene expression in adipocytes. *Journal of Lipid Research*.

[23] Feng X., Zhang L., Xu S., Shen A. Z. (2020). ATP-citrate lyase (ACLY) in lipid metabolism and atherosclerosis: an updated review. *Progress in Lipid Research*.

[24] ALJohani A. M., Syed D. N., Ntambi J. M. (2017). Insights into stearoyl-CoA desaturase-1 regulation of systemic metabolism. *Trends in Endocrinology and Metabolism*.

[25] Pham T., Walden E., Huard S., Pezacki J., Fullerton M. D., Baetz K. (2022). Fine-tuning acetyl-CoA carboxylase 1 activity through localization: functional genomics reveals a role for the lysine acetyltransferase NuA4 and sphingolipid metabolism in regulating Acc1 activity and localization. *Genetics*.

[26] Aguiló F., Camarero N., Relat J., Marrero P. F., Haro D. (2010). Transcriptional regulation of the human acetoacetyl-CoA synthetase gene by PPARgamma. *The Biochemical Journal*.

[27] Moffett J. R., Puthillathu N., Vengilote R., Jaworski D. M., Namboodiri A. M. (2020). Acetate revisited: a key biomolecule at the nexus of metabolism, epigenetics and oncogenesis-part 1: acetyl-CoA, acetogenesis and acyl-CoA short-chain synthetases. *Frontiers in Physiology*.

[28] Chen C. C., Hsu L. W., Huang K. T., Goto S., Chen C. L., Nakano T. (2017). Overexpression of Insig-2 inhibits atypical antipsychotic-induced adipogenic differentiation and lipid biosynthesis in adipose-derived stem cells. *Scientific Reports*.

[29] Mori E., Fujikura J., Noguchi M. (2016). Impaired adipogenic capacity in induced pluripotent stem cells from lipodystrophic patients with *BSCL2* mutations. *Metabolism*.

[30] Kong S., Cai B., Nie Q. (2022). PGC-1*α* affects skeletal muscle and adipose tissue development by regulating mitochondrial biogenesis. *Molecular Genetics and Genomics*.

[31] Walton R. G., Zhu B., Unal R. (2015). Increasing Adipocyte Lipoprotein Lipase Improves Glucose Metabolism in High Fat Diet-induced Obesity. *The Journal of Biological Chemistry*.

[32] Chedid M. F., do Nascimento F. V., de Oliveira F. S. (2019). Interaction of HSD11B1 and H6PD polymorphisms in subjects with type 2 diabetes are protective factors against obesity: a cross-sectional study. *Diabetology and Metabolic Syndrome*.

[33] Yamauchi T., Iwabu M., Okada-Iwabu M., Kadowaki T. (2014). Adiponectin receptors: a review of their structure, function and how they work. *Best Practice & Research. Clinical Endocrinology & Metabolism*.

[34] Qin D. D., Yang Y. F., Pu Z. Q. (2018). NR4A1 retards adipocyte differentiation or maturation via enhancing GATA2 and p53 expression. *Journal of Cellular and Molecular Medicine*.

[35] Stefanovic V., Antic S. (2004). Plasma cell membrane glycoprotein 1 (PC-1): a marker of insulin resistance in obesity, uremia and diabetes mellitus. *Clinical Laboratory*.

[36] Kitao N., Nakamura A., Miyoshi H. (2018). The role of glucokinase and insulin receptor substrate-2 in the proliferation of pancreatic beta cells induced by short-term high-fat diet feeding in mice. *Metabolism*.

[37] Cahua-Pablo J. A., Flores-Alfaro E., Cruz M. (2016). Estrogen receptor alpha in obesity and diabetes. *Revista Médica del Instituto Mexicano del Seguro Social*.

[38] Ji X., Li N., Yang R., Rao K., Ma M., Wang Z. (2020). The steroid receptor coactivator 1 (SRC1) and 3 (SRC3) recruitment as a novel molecular initiating event of 4-n-nonylphenol in estrogen receptor *α*-mediated pathways. *Ecotoxicology and Environmental Safety*.

[39] Greenhill C. (2020). ER*α* affects mitochondrial function in adipocytes. *Nature Reviews. Endocrinology*.

